# Treatment of La Grippe

**Published:** 1890-02

**Authors:** 


TREATMENT OF LA GRIPPE.—If the attack occurs in the morning a mild cathartic is administered, generally calomel in from two to ten-grain doses. This is followed at night by a ten-grain Dover's powder and ten grains of quinine. On the following day, when the catarrhal symptoms become marked, a pill of morphine (gr. 1-10), ext. bellad. (gr. one-half), camph. (gr. one-half), and quin. (two or three grs.), is given every three or four hours.

When the bronchitis is the prominent feature, a mild expectorant mixture is given, containing syr. licorice, squills, senega, chloride of ammonia, morphine, etc. For frontal headache antypirin, ten to fifteen grains, with whisky, is administered.—*Med. Record.*

